# Using sibling data to explore the impact of neighbourhood histories and childhood family context on income from work

**DOI:** 10.1371/journal.pone.0217635

**Published:** 2019-05-30

**Authors:** Lina Hedman, David Manley, Maarten van Ham

**Affiliations:** 1 Center for Research and Development, Uppsala University/Region Gävleborg, Gävle, Sweden; 2 Institute for Housing and Urban Research, Uppsala University, Uppsala, Sweden; 3 Department of Urbanism, Faculty of Architecture and the Built Environment, Delft University of Technology, Delft, The Netherlands; 4 School of Geographical Sciences, University of Bristol, Bristol, United Kingdom; 5 School of Geography and Sustainable Development, University of St Andrews, St Andrews, United Kingdom; University of Jyvaskyla, FINLAND

## Abstract

Previous research has reported evidence of intergenerational transmissions of neighbourhood status and social and economic outcomes later in life. Research also shows neighbourhood effects on adult incomes of both childhood and adult neighbourhood experiences. However, these estimates of neighbourhood effects may be biased because confounding factors originating from the childhood family context. It is likely that part of the neighbourhood effects observed for adults, are actually lingering effects of the family in which someone grew up. This study uses a sibling design to disentangle family and neighbourhood effects on income, with contextual sibling pairs used as a control group. The sibling design helps us to separate the effects of childhood family and neighbourhood context from adult neighbourhood experiences. Using data from Swedish population registers, including the full Swedish population, we show that the neighbourhood effect on income from both childhood and adult neighbourhood experiences, is biased upwards by the influence of the childhood family context. Ultimately, we conclude that there is a neighbourhood effect on income from adult neighbourhood experiences, but that the childhood neighbourhood effect is actually a childhood family context effect. We find that there is a long lasting effect of the family context on income later in life, and that this effect is strong regardless the individual neighbourhood pathway later in life.

## Introduction

There is an emerging body of literature that highlights the importance of taking into account the neighbourhood in which an individual grew up as a means to understand their later in life trajectories. Empirical evidence suggests that there is a correlation between the neighbourhood types experienced during childhood and the neighbourhoods where one lives in adulthood [1,2,3,4,5,6]. Other studies show that the neighbourhood environment experienced during childhood has a causal and long-lasting influence on adulthood socio-economic status outcomes, such as income [7,8,9,10,11,12]. The size of the effects of the childhood neighbourhood on individual outcomes is unclear, both in absolute and relative terms. When reviewing literature on neighbourhood effects on children’s outcomes, Ginther and colleagues [13] found effects which varied from substantial to almost non-existent. They argued that one explanation for these disparities could be that models of neighbourhood effects often do not control for family characteristics, which can result in biased outcomes. There are some examples of studies which argue that neighbourhood effects are very small or even non-existent when taking the family context into account (for instance see [14]). It is notable that family effects on children’s outcomes are generally found to be substantially higher than neighbourhood effects [15].

This paper contributes to the discussion on the relative importance of childhood and adulthood neighbourhood experiences in explaining later-in-life income from work, by explicitly taking the childhood family context into account. To disentangle family and neighbourhood context effects, we employ a sibling design in which the incomes of full siblings are compared. Full siblings share a substantial part of their genes, and are often raised under similar circumstances. They also share childhood neighbourhood histories and, importantly, parental motivations for moving to certain neighbourhoods. This implies that by using a sibling design, any potential selection effects related to the family’s entry into the childhood neighbourhood are effectively negated. We compare outcomes for full siblings to a control group of what we call ‘contextual siblings’; these are unrelated individuals who we randomly paired together, but who share their childhood neighbourhood. Comparisons between the two groups allow us to distinguish between effects on later-in-life income that are due to family context or childhood neighbourhood context. Contrary to much previous work on neighbourhood effects, but in line with the (Scandinavian) economic literature using sibling comparisons, we find that the childhood neighbourhood has only a very limited effect on future income whereas the family context plays a major role.

## Neighbourhood and family effects

Neighbourhood effects arise due to critical spatial context exposures that affect individual life opportunities through a set of transmission mechanisms [16]. Although the residential neighbourhood does not represent the full range of exposures that an individual experiences [17], it acts like an access point through which many other contextual spaces are accessed. Hence, geographic variation in the local spatial opportunity structure [18] not only concerns the neighbourhood but also higher geographic levels in which the neighbourhood is situated within (for example, school attachment areas, city districts, the municipality etc.). There is a vast literature analysing how neighbourhood exposures affect individual life opportunities. This literature includes outcomes such as indicators of socio-economic status, school performance, health, cognitive abilities, behaviours etc., and encompasses studies from different countries and cities, using different methodological approached and data sets, as well as varying neighbourhood definitions. Most of these studies find evidence of neighbourhood effects (there are however also examples of studies finding no effects at all; see [19, 20, 21]). Studies have also found neighbourhood effects to vary by individual characteristics [22, 23], spatial scale [24, 25] and length of exposure to certain neighbourhood types [23, 26, 27].

The issue of timing of exposure has also been found to be central in understanding neighbourhood effects. Using experimental data from the Moving to Opportunity programme, Chetty and colleagues [7] demonstrate that moving from a high- to a lower-poverty area before the age of 13 is associated with increased college attendance, and higher earnings and lower risks of single parenthood later in life. It should, however, be noted that the scale of neighbourhood used by Chetty and colleagues was far greater than is usually deployed in the neighbourhood effects literature. Similarly, Galster and Santiago [8] find that children perform better (measured at age 18) if being exposed to higher-performing neighbours at a younger age. The results by Chetty et al and Galster and Santiago suggest that at least part of the neighbourhood effects are temporally lagged and long lasting (see also [11, 12, 28]). This is confirmed in a study by Hedman and colleagues [10], who find for Sweden that the parental neighbourhood affects the incomes of children up to at least 17 years after leaving the parental home. A study by Sharkey and Elwert [29] suggests that children’s cognitive ability is influenced by the neighbourhood of their parents, even though the child has never lived in the area him/herself. This transmission is suggested to operate through long lasting effects on parents which are then affecting the outcomes of their children.

Sharkey and Elwert [29] argue that their finding of ‘multigenerational effects’ provides evidence of multigenerational neighbourhood effects. Studies from both sides of the Atlantic have reported multigenerational continuity in the neighbourhood environment; children living with their parents in deprived areas are more likely to reside in similarly deprived neighbourhoods as adults than others [2, 3, 4, 5, 6]. This literature argues that the neighbourhood environment is transmitted across generations in a similar way to other features of socio-economic status, via mechanisms such as inherited financial opportunities, transmission of norms and values, transmission of housing preferences and restrictions common to both parents and children (for example, belonging to a minority group). Hence, the choice (or lack thereof) of neighbourhood in adulthood is affected by both childhood neighbourhood experiences and the childhood family context. This conclusion was confirmed by Manley and colleagues [30] who compare residential neighbourhood careers of siblings to unrelated individuals originating from the same neighbourhoods. In accordance with previous literature, they find that neighbourhood status is reproduced over time, but add that siblings live more similar lives (in terms of neighbourhood environment) than unrelated individuals. The authors suggest that this is due to siblings’ shared family context experiences, which in turn influences their future neighbourhood choices.

The findings of long-lasting (from childhood through to adulthood), and multigenerational neighbourhood effects and transmission of neighbourhood status, suggest that it is difficult to separate neighbourhood context effects from family context effects. Both the neighbourhood of residence experienced in adulthood and adult income seem to be directly and indirectly influenced by parental choices and characteristics. Galster and colleagues [9] illustrate this interconnectedness with a “holistic framework” in which they suggest that outcomes of young adults are determined by individual characteristics (both observed and unobserved) and parental characteristics (both observed and unobserved) and that those parental characteristics that remain unobserved may lead to selection biases. Specifically, parental income or wealth, together with a number of other attributes (such as network, cognitive resources, and potentially restricting characteristics), determine the range of neighbourhoods available to them and hence the neighbourhood environments experienced by their children. The same family context variables are also known to influence children’s socio-economic outcomes, which makes it difficult to separate neighbourhood effects from family context effects.

A large literature has documented intergenerational similarities in socio-economic characteristics between parents and their children (father-son income correlations are especially common; see [31, 32, 33, 34]). This literature has become increasingly engaged with explaining such intergenerational patterns. The literature provides a number of factors which might explain how parents influence their children’s adulthood socio-economic status. The first is related to parental resources. Parental education and income are often regarded as the most important explanations of socio-economic outcomes for children (see [33]). These parental resources are also known to affect children’s health-awareness, fertility behaviour (timing and number of children), demand for cultural goods and services, and attitudes towards work and education. Another factor influencing intergenerational transmission is that the family context can expose children to various social problems, including exposure to violence, drug and alcohol abuse, criminality and mental illness. Such exposures may affect children’s outcomes through transmittance of behaviour (where parents act as role models) or developmental problems. A third broad factor is the household environment, including family structure, parental style and norms and values. Empirical evidence suggest that growing up in a single-parent household or in a large family is positively associated with school drop-out and future unemployment (e.g. [35, 36]). Björklund and colleagues [14] test the effects of a number of different family traits and find that parental involvement in children’s school work, parental firmness, maternal patience (willingness to plan ahead and postpone financial gains), and the number of books in the parental home are all significant factors explaining the ‘family effect’ when comparing incomes of siblings. Mason [37] argues that intergenerational transmission of family norms and values are significantly related to children’s future socio-economic outcomes, controlling for parents’ socio-economic status. Norms, attitudes and values can be transmitted through a learning process within the family, but behaviours or attitudes may also be affected by genetic composition which obviously is (partially) transmitted between parents and their (biological) offspring. Genetic composition has been demonstrated to affect, among other things, cognitive abilities, personality traits [38, 39] and risk-taking behaviour [40] which all are likely to affect future income levels.

## The usage of siblings to separate family and neighbourhood effects

Using a sibling design has been argued to be a promising approach to separate neighbourhood context effects from family context effects, although such a design is not used very often [14]. Within pairs of genetically related individuals, who also share a similar family background (siblings), many of the unmeasured influences on individual outcomes can be controlled for. If siblings are sufficiently close in age, they will have experienced a similar household environment, and it can be assumed that they have also been exposed to the same family norms, values and attitudes. They will also have similar childhood neighbourhood experiences, at least in terms of their residential locations. Any sibling correlation can thus be assumed to represent a joint effect of shared family and community characteristics [34]. Sibling correlations in income are generally found to be about 0.45 for the U.S and 0.25 for the Scandinavian countries [31]. This means that, in the U.S., almost half of the inequality in earnings can be attributable to siblings’ shared background.

To separate family and neighbourhood effects, an outcome variable can be decomposed into a family and a neighbourhood component, where the first is based on sibling comparisons and the second on comparing neighbouring but unrelated children. Studies using such a design generally find that the neighbourhood context is relatively unimportant, at least in comparison with the family context. Several U.S. and U.K based studies find neighbourhood correlations in the range of 0.1–0.2 while family correlations tend to be at least twice as high [15, 41, 42, 43]. Using Swedish data for 13,000 individuals born in 1953, Lindahl [44] estimates the relative significance of family and childhood neighbourhood for school performance, educational attainment and income. Lindahl finds sibling correlations to take on values between 0.17 (income, females) and 0.43 (education, females), whereas the highest neighbourhood correlation found, unadjusted for parental background characteristics, was below 0.08 (education, males). Adjusted for parental background characteristics, neighbourhood correlations dropped to below 0.03. Equally weak neighbourhood correlations (well below the levels of the U.S. and U.K.) have been reported by Brännström [45] for Sweden, and for other countries such as Norway [46], and for Toronto, Canada [47]. These studies nuance the findings of many neighbourhood effects studies which do not take the family context into account.

A different methodological approach that has been widely used for sibling designs is the family-fixed effects modelling framework. As noted previously, a key challenge within the neighbourhood effects literature is to remove bias due to (own or parental) sorting. This is commonly achieved by using fixed-effects. By combining fixed-effects with a sibling design, it is possible to difference out all time-invariant family-related unobservable characteristics that would otherwise risk biased estimates, if correlated with both the residential sorting of families and the outcome of choice (see Aaronson 1998 for a more thorough description). Family-fixed effects are also common in studies of intergenerational social mobility, where the aim is to isolate causal influence of the family (rather than neighbourhood) on outcomes like income or school performance. Aaronson [48] uses family fixed-effects for a sample of U.S. siblings from the Panel Study of Income Dynamics (PSID) and finds some evidence of neighbourhood effects when controlling for family-specific unobservables. Using the same data source, Vartanian and Walker Buck [49] model childhood and adolescence neighbourhood effects on adult income. Like Aaronson, they find evidence of such effects for children of all age groups. Even very young children (aged 0–4) would, according to Vartanian and Walker Buck, benefit in terms of future incomes from living in more advantageous neighbourhood environments. Contrary to these results, Plotnick and Hoffman [20] find neighbourhood effects only when controlling for family observed variables but not when using the family fixed-effect approach, which also removes family-related unobservables. Like Aaronson and Vartanian and Walker Buck, they base their study on PSID data but they restrict their sample to young adult women. Plotnick and Hoffman conclude that selection due to unobservable factors is really important and warrants caution, while also stating the possibility that their results are sample-specific. However, these studies suffer from a shortcoming that is recognized by both the authors and critical readers: by requiring variation in neighbourhood exposure between siblings, obtained through selecting siblings of different ages, gender or any other key characteristic, the design risks violating the assumption of similarity in family background. Siblings born further apart have a higher risk of being raised under different circumstances. If the unobservable family characteristics are correlated with sibling differences in neighbourhood exposure, and also affect the dependent variable (Aaronson [48] mentions ability, ambition and parental expectations as potential such candidates), the ‘family effect’ will not be completely removed.

Sibling designs have also been used to isolate neighbourhood effects within the field of health. One example is a study by Merlo and colleagues [50] in which they analyse the relationship between (adult) neighbourhood exposure and the risk of ischemic heart disease. They use a dataset of Swedish-born brothers and calculate average exposure to low-income neighbourhoods for each pair of brothers, intended to capture the brothers’ joint exposure. To capture individual trajectories, they also calculate how each individual departures from this joint family mean. Both these variables (family mean and individual departure) are used to estimate the relative impact of family and adult neighbourhood trajectory using a multilevel modelling strategy. The authors conclude that the intra-family correlation is much higher than the intra-neighbourhood correlation. In fact, they find that the latter is very small, in the order of 1.5%. They also show that the estimated neighbourhood effect gets much smaller when taking the exposure of the full brother into account.

In the current paper we adopt an analytical strategy which is similar to Merlo and colleagues [50]. We analyse the residential trajectories of siblings within a multilevel framework, and we calculate joint sibling exposure and individual departures, and compare these results to an ‘individual model’ where the residential trajectory is based solely on individual experiences. Unlike Merlo and colleagues, but in line with the literature on intergenerational social mobility, we also compare the siblings to a group of randomly paired unrelated individuals, but who originate from the same neighbourhood; which we call ‘contextual siblings’. Using this design we are able to isolate the part of the variation in the outcome variable that is due to family context and childhood neighbourhood context, while also getting estimates for the effects of exposure of the adulthood neighbourhood trajectory.

## Data and methods

This paper is part of a project funded by the European Research Council (ERC). As part of the granting procedure, the project proposal was evaluated by both the Delft University of Technology institutional ethics committee, and the ERC ethics committee. Both committees approved the project. The data used for this study are derived from GeoSweden, a register based longitudinal individual level micro-database owned by the Institute for Housing and Urban Research, Uppsala University. The GeoSweden database is not based on a sample, but it contains the entire Swedish population tracked from 1990 to 2010. The database is constructed from a set of different annual administrative registers including, demographic, geographic, socio-economic and real estate data for each individual living in Sweden each year. For each person in the dataset it is possible to identify their parents and through them also their siblings. Although the data used cannot be publicly shared, we have made our Stata code available through protocols.io to enhance the reproducibility of our research (http://dx.doi.org/10.17504/protocols.io.z6af9ae).

Contrary to most previous neighbourhood effect studies using siblings, we are explicitly interested in any long-lasting effects from childhood neighbourhoods on adulthood outcomes. Since the dependent variable is (logged) income from work, including work-related transfers, we need to follow individuals for a sufficiently long time to move beyond the most turbulent years (at the beginning of the labour market career of individuals) where incomes tend to fluctuate. Income from work represents the sum of cash salary payments, income from active businesses, and tax-based benefits that employees accrue as terms of their employment (sick or parental leave, work-related injury or illness compensation, daily payments for temporary military service, or giving assistance to a handicapped relative). For both siblings to have reached a more stable stage in life within the 20-year period for which data are available, we need them to leave the parental home in the beginning of the data period. In practice, we have selected siblings that leave the parental home between 1990 and 1997 (the year of departure we denote as *t*), which allow us to follow all individuals for 14 consecutive years. The dependent variable, logged income from work, is measured as the average income in years 12, 13 and 14 post leaving the parental home, to reduce biases due to temporary fluctuations. Since the calendar years of these events (year *t*+12 –*t*+14) vary among individuals, we have adjusted income for inflation with 1990 as a base year. This leaves us with eleven years over which we can follow individual neighbourhood trajectories (to avoid spurious correlation, we begin measuring the dependent variable one year after we measure the independent ones. Hence, all independent variable are measured at t+11). Our individuals are then around 30 years of age, an age when most should have finished their studies, established themselves on the labour market, settled down and are likely to have started or be starting a family.

We measure neighbourhood exposure in adulthood rather than in childhood (as is done in much previous work). Since both siblings have left the parental home during our period of study, we obtain the necessary neighbourhood variation within sibling pairs almost by default. It is very unusual for two siblings to live in the same neighbourhood environment for eleven consecutive years of independent housing careers. Hence, there is no need to select siblings on the criterion of variation in childhood neighbourhood exposure—rather the contrary since childhood neighbourhood and childhood family variation are associated and the basic idea of the sibling setup is to identify individuals with similar family exposures. Unlike much previous work, we thus select siblings on the criterion of *similarity*. Only if the siblings are sufficiently similar can we argue that they share family exposure which consequently can be controlled away when comparing later-in-life outcomes of the siblings.

To be included in the data, siblings must meet all the following criteria: i) both siblings are aged between 15–21 in 1990; ii) siblings are born no more than three years apart; iii) both siblings live in the parental home in 1990; iv) at least one of the siblings leaves the parental home between 1991 and 1993; v) the other sibling leaves the parental home at most four years after the first sibling; vi) the siblings are of the same sex. The parental home could be either the mother’s or the father’s home, as long as both siblings live in the same home. For simplicity, we have restricted the analysis to two siblings per family. In case of multiple sibling pairs within the same family that fulfil the above criteria, we have selected the sibling pair closest in age to maximise similarity of exposure to neighbourhood and family environment and resources. If there were multiple sibling pairs within the same family with the same age difference, we have selected the oldest pair. Analyses are run separately by gender. The above described restrictions have left us with 19,706 males (9,853 male sibling pairs), and 24,924 females, (12,462 female sibling pairs). We acknowledge that the matching process used in the data design is relatively simplistic. We have adopted this approach rather than a more sophisticated approach for both pragmatic and conceptual reasons. Pragmatically, the group of contextual siblings is already substantially smaller than the group of real siblings, and further restrictions risk reducing the group even further. Conceptually, important elements of the family relationship such as genetic similarity and the precise nature of the exposure to family environments and behaviours, is unmeasured anyway, and much of this is already accounted for in the fixed part of our model.

Neighbourhoods are defined according to the SAMS (Small Area Market Statistics) classification scheme, made by Statistics Sweden in collaboration with each respective municipality. The SAMS areas are constructed to be relatively homogenous in terms of housing type, tenure and construction period. Although the usage of administrative areas in neighbourhood effect studies has been critiqued [51], we argue that SAMS areas capture the physical structure of the surrounding environment sufficiently well, and are often used in similar research and maintaining this approach allows our results to be comparable. More importantly, bespoke neighbourhoods (see [24]) are inappropriate here because we need fixed neighbourhood boundaries to be able to construct a control group (the contextual sibling pairs, described later). Our neighbourhood variable of interest is the share of low income individuals in each neighbourhood. We define low income as belonging to the three lowest income deciles based on the national income distribution. For each year and neighbourhood in the data, we calculate the share of low-income earners based among working-age people (20–64), for neighbourhoods with at least 30 inhabitants in working ages. As noted above, all independent variables in our analyses are measured eleven years after having left the parental home. Unlike the other independent variables, neighbourhood exposure is measured cumulatively, over the period 1–11 years after leaving the parental home. As a consequence of this cumulative measure, the neighbourhood variable can, theoretically, take values between 0 and 1100 (where the value 1100 equates to exposure to low-income neighbours for the entire eleven year period). We have not included characteristics of the childhood neighbourhood into our models, and we only provide the childhood neighbourhood variation (see next paragraph on modelling strategy). The reason for not including childhood neighbourhood characteristics in our models is related to the risk of overcontrolling our models. It is likely that childhood neighbourhood characteristics also affect household status later in life, level of education, employment status, housing tenure, and residential neighbourhood trajectory. By controlling for these characteristics, and then childhood neighbourhood characteristics as well, the potential range of neighbourhood effects on income is vastly truncated.

### Modelling strategy

We model neighbourhood effects using a multilevel framework: individuals nested in families, nested into childhood neighbourhoods. We adopt this approach given that we wish to identify if the childhood neighbourhood has a lasting impact on later life outcomes (see [28]) and to recognise the clustering at the level of the childhood neighbourhood in the data. The multilevel model provides us with a tool to separate family level variation from childhood neighbourhood variation. Thus, the model setup allows us to take a first step towards identifying a neighbourhood effect that is not confounded by the family context.

The model is written as:

$$\ln\left({inc}_{ijk}\right)=\alpha +{\beta 1X}_{ijk}+{\beta 2Y}_{ijk}+{\beta 3N}_{ijk}+v_{k}+\mu_{jk}+e_{ijk}$$ (1)

Where:

ln(*inc*_*ijk*_) = logged income from work, including work-related benefits, measured as the average over years 12–14 after leaving the parental home

X_*ijk*_ = a range of individual control variables that are time-invariant or not affected by family (age, sex, country of birth)

Y_*ijk*_ = a range of individual control variables that are time-variant and might be affected by childhood family or childhood neighbourhood (household composition, education level, employment status, tenure), all measured 11 years after leaving the parental home

*N*_*ijk*_ = individual cumulative neighbourhood exposure, measured over the period from leaving the parental home and 11 years onward

*v*_*k*_ = variation at the childhood neighbourhood level

*μ*_*jk*_ = variation at the family level

*e*_*ijk*_ = an individual error term

In order to fully benefit from the sibling relationship in our data, we adopt the strategy of Merlo and colleagues [50] and compare the ‘standard model’ as described above, and which measures neighbourhood exposure at the individual level, to a model where the individual estimate is replaced by two variables. The first of these is estimated at the family level: *family mean of cumulative neighbourhood exposure* and the second variable measures the *individual departure from the family mean*. The family mean of cumulative neighbourhood exposure represents the average of adult neighbourhood exposures of the two siblings. Given that the variable takes the neighbourhood pathways of both siblings into account, it implicitly contains familial background aspects shared by siblings that affect their residential paths. Thus, although we cannot directly measure these shared aspects, the family mean variable may well capture effects of shared genetic composition, abilities, temperament, upbringing, norms and values, attitudes, parental guidance, (monetary) support or other tangible and intangible items shared by siblings but not by unrelated individuals.

The second variable, *individual departure from family mean*, is obtained by subtracting the family mean from the individual exposure. Hence, the variable estimates the extent to which the individual pathway deviates from the shared sibling exposure. A positive value means that the individual has a higher exposure to low income neighbours over the last eleven years than their sibling. We argue that by replacing individual neighbourhood exposure by these two variables, family mean and individual departure from the family mean, we are able to distinguish the family influence from the neighbourhood effect arising from adulthood neighbourhood exposure. Any lingering family influence that affects both siblings similarly should be captured by the family mean whereas the individual departure variable represents the unique pathway of each individual, free from family influence.

Thus, our model using these two variables is written as:

$$\ln\left({inc}_{ijk}\right)=\alpha +{\beta 1X}_{ijk}+{\beta 2Y}_{ijk}+{\beta 4F}_{jk}+{\beta 5I}_{ijk}+v_{k}+\mu_{jk}+e_{ijk}$$ (2)

In Eq 2, the individual cumulative neighbourhood exposure, N_ijk_, has been replaced by the two variables, family mean of cumulative neighbourhood exposure, F_jk_, and individual departure from the family mean, I_ijk_.

As part of our modelling design we create a control group consisting of a set of what we term ‘contextual sibling pairs’. These are synthetic pairs who originate from the same neighbourhood and should hence share any advantages (or disadvantages) arising from the place in which they grew up. However, unlike real siblings, they do not share parents, so their family-based upbringing, genes and other family factors differ. The contextual sibling pairs are created by selecting all individuals in the same age range as the ‘real’ siblings (15–21 in 1990) and ordering them randomly by neighbourhood of origin, father’s country background (Sweden, West, Eastern Europe incl. Russia or Non-western countries), and father’s income level. We then subject the contextual sibling pairs to the same restrictions as our real siblings: 1) they should be born no more than three years apart; 2) at least one should leave the parental home between 1991 and 1993; 3) they should leave home a maximum of four years apart. All pairs not fulfilling these criteria are deleted. We also delete any real sibling pairs, deriving from either the father or the mother. The randomly paired up individuals are fewer in numbers than the real siblings: 8,300 individuals in 4,150 pairs.

If our modelling approach functions properly, the ‘family’ level variance for the contextual siblings should be close to zero (since they are not related variation here would be erroneous). In addition, we expect that the estimates of the ‘family’ mean and individual departure from the ‘family’ mean variables behave differently. The ‘family’ mean of the contextual siblings should not capture anything else than a simple mean of the neighbourhood paths of two unrelated individuals. We hypothesize that unrelated individuals (the contextual siblings) will experience more variation in terms of their adult neighbourhood paths (since there is no shared family influence that might affect their future neighbourhood choices). As a result, the ‘family’ mean variable will matter less for the contextual siblings compared to the actual sibling pairs. The individual departure from the ‘family’ mean variable will, however, likely to be more important for the set of contextual siblings since this variable captures the individual pathway and its influence on work income.

As individual controls, we include variables that are time-invariant and/or unaffected by childhood family or childhood neighbourhood, including age, sex (in the models using mixed pairs) and father’s country of birth (Sweden, Western countries, Eastern Europe incl. Russia, Non-western countries). We have chosen to define country of birth through the father since children of immigrants often face similar difficulties as their first-generation immigrant parents, and because many children bear their father’s family name which is a strong marker of ethnicity. We also include a range of control variables that are likely to be endogenous as they are causally related to our independent variable, income from work, but also probably affected by family and/or childhood neighbourhood factors. These are partner status (single or living with a partner), whether there are children in the household (no or yes), level of education measured in years (categorised as under 12 years, 12 years, 13–14 years, or 15+ years), employment status (in paid employment or not), and housing tenure (home ownership, tenant-owned cooperative, rental). Not including these control variables in the analysis would risk overestimating the effect of the adult neighbourhood path on income from work. However, if we include these variables, we risk *underestimating* the true neighbourhood effect. Unfortunately, there is no easy way around this problem so we address it by running our models both including and excluding the endogenous time-variant control variables. Thus, whilst we cannot obtain an exact measure of any ‘true’ neighbourhood effect arising from adulthood neighbourhood exposure, we can estimate an interval within which any effect is likely to be found. We find this to be a useful solution and one that avoids providing certainty around a statistical estimate which is anything but. Descriptive statistics of all variables included in the analysis is shown in Table 1.

**Table 1:** Descriptive statistics.

			Real siblings								Synthetic siblings							
			Males				Females				Males				Females			
*Continuous variables*		*Time of measurement*	*Mean*	*Std Dev*	*Min*	*Max*	*Mean*	*Std Dev*	*Min*	*Max*	*Mean*	*Std Dev*	*Min*	*Max*	*Mean*	*Std Dev*	*Min*	*Max*
Log of income, money value 1990		average of *t*+12—*t*+14	7.04	2.02	0.00	10.40	6.25	2.44	0.00	9.08	7.56	0.66	2.39	9.54	7.14	0.73	0.78	8.74
Cumulative % low-income neighbours		*t*—*t*+11	349.56	89.69	50.24	969.02	340.85	81.90	56.52	940.84	348.96	89.76	141.06	856.34	338.11	78.68	54.38	757.57
Family mean cum % low-income neighbours		*t*—*t*+11	349.55	75.16	142.66	888.81	340.84	68.26	147.01	824.14	349.02	67.96	179.29	646.94	338.13	61.02	171.23	637.02
Individual departure from family mean		*t*—*t*+11	0.00	48.92	-343.43	401.81	0.01	45.25	-304.14	304.14	-0.06	58.71	-264.52	264.52	-0.01	49.69	-236.04	236.04
Age		*t*+11	31.20	1.57	25.00	38.00	30.55	1.53	25.00	38.00	31.47	1.65	26.00	38.00	30.73	1.60	26.00	37.00
***Categorical variables***			*** ***	*** ***	*** ***	*** ***	*** ***											
Father's country of birth	Sweden	*t*+11	90.40				90.24				93.83				94.91			
	West	*t*+11	6.47				6.32				4.46				3.36			
	East	*t*+11	1.22				1.50				0.80				0.48			
	Non-west	*t*+11	1.92				1.93				0.91				1.25			
Live with partner	single	*t*+11	47.30				37.27				46.46				34.49			
	partner	*t*+11	52.70				62.73				53.54				65.51			
Children in household	no	*t*+11	52.41				33.45				51.03				31.03			
	yes	*t*+11	47.59				66.55				48.97				68.97			
Education level	LT 12 years	*t*+11	44.64				32.84				41.60				29.20			
	12yrs	*t*+11	18.43				24.57				19.37				23.78			
	13-14yrs	*t*+11	15.83				15.83				16.86				16.57			
	15+yrs	*t*+11	21.10				26.76				22.17				30.45			
Employed	not employed	*t*+11	9.90				17.49				6.69				11.91			
	employed	*t*+11	90.10				82.51				93.31				88.09			
Tenure	home ownership	*t*+11	46.66				52.49				49.83				54.27			
	tenant-based coop	*t*+11	19.76				15.85				18.40				15.42			
	rental	*t*+11	33.59				31.66				31.77				30.31			
N			19706				24924				1750				2082			

## Results

Table 2 shows the results from our ‘individual models’–the models estimating the effect of adult neighbourhood exposure at the level of individuals on income for work, separately for male and female same-sex siblings. Models 2a and 2c includes only characteristics that are not influenced by parents/childhood neighbourhood (age and country of birth). In Models 2b and 2d we add time-varying variables that are known to affect income from work (family composition, education level, employment status, tenure), but which are also highly likely to be influenced by childhood family context and childhood neighbourhood exposure.

**Table 2:** Results for real siblings, from individual models, using own cumulative exposure to poverty neighbourhoods. Dependent variable = logged income from work.

		Males								Females							
		Model 2a				Model 2b				Model 2c				Model 2d			
		Coef.	Std. Err.	Conf interval 95%		Coef.	Std. Err.	Conf interval 95%		Coef.	Std. Err.	Conf interval 95%		Coef.	Std. Err.	Conf interval 95%	
**Predictor variables**																	
Total sum % low-income neighbours over 11 years		-0.0021	0.0002	-0.0024	-0.0018	-0.0012	0.0001	-0.0015	-0.0009	-0.0020	0.0002	-0.0024	-0.0016	-0.0013	0.0002	-0.0017	-0.0010
Age		0.0804	0.0091	0.0626	0.0982	0.0127	0.0074	-0.0018	0.0272	0.0129	0.0101	0.1089	0.1484	0.0113	0.0088	-0.0059	0.0286
Father's country of birth (ref = Sweden)	West	-0.3258	0.0618	-0.4470	-0.2047	-0.0741	0.0474	-0.1670	0.0189	-0.3042	0.0665	-0.4346	-0.1738	-0.0829	0.0546	-0.1899	0.0241
	East	-0.8858	0.1387	-1.1576	-0.6139	-0.3991	0.1063	-0.6074	-0.1907	-0.3886	0.1334	-0.6501	-0.1271	-0.1526	0.1093	-0.3669	0.0617
	Non-west	-1.1877	0.1124	-1.4080	-0.9674	-0.6209	0.0867	-0.7909	-0.4509	-1.6598	0.1205	-1.8960	-1.4236	-0.6980	0.0997	-0.8935	-0.5026
Live with partner (ref = single)						0.0627	0.0434	-0.0224	0.1478					0.0260	0.0393	-0.0510	0.1030
Children in household (ref = no))						0.1270	0.0434	0.0419	0.2122					-0.0805	0.0388	-0.1565	-0.0046
Education level (ref = LT12yrs)	12yrs					0.2298	0.0316	0.1679	0.2917					0.2870	0.0349	0.2186	0.3554
	13-14yrs					0.4592	0.0338	0.3929	0.5255					0.5405	0.0406	0.4610	0.6201
	15+yrs					0.6799	0.0323	0.6166	0.7432					0.8487	0.0364	0.7774	0.9201
Employed (ref = not employed)						3.8144	0.0391	3.7378	3.8910					3.1925	0.0349	3.1241	3.2610
Tenure (ref = home ownership)	cooperative					-0.0569	0.0333	-0.1222	0.0083					-0.0560	0.0398	-0.1340	0.0220
	rental					-0.1151	0.0299	-0.1736	-0.0565					-0.1312	0.0344	-0.1986	-0.0638
Constant		5.3205	0.2945	4.7433	5.8978	3.3392	0.2415	2.8658	3.8126	3.0610	0.3198	2.4343	3.6878	3.4490	0.2782	2.9036	3.9943
**Random effects parametres**																	
Childhood neighbourhood variance		0.0187	0.0194	0.0024	0.1434	0.0000	0.0000	0.0000	0.0000	0.0261	0.0222	0.0049	0.1381	0.0000	0.0000	0.0000	0.0000
Family variance		0.5572	0.0445	0.4766	0.6516	0.1153	0.0258	0.0743	0.1788	0.6706	0.0566	0.5684	0.7911	0.2109	0.0374	0.1490	0.2987
Residual		3.3818	0.0483	3.2884	3.4778	2.4236	0.0347	2.3566	2.4926	5.1134	0.0649	4.9877	5.2422	3.9414	0.0501	3.8445	4.0408
N		19706				19706				24924				24924			
Log Likelihood		-41410.782				-37132.004				-57202.893				-53091.455			

The share of the variation that can be attributed to the three levels (individual, family and neighbourhood) is instructive. Models 2a and 2c show that for both males and females, only a very small part of the variation in later-in-life income is related to the childhood neighbourhood. Given that we measured income as the average income from work over years 12–14 after having left the parental home and neighbourhood, it is not surprising that the effect of the childhood neighbourhood is low. However, the effect is still present. Variation at the family level is considerably higher and corresponds to between 12% (females) and 14% (males) of the total variation in income. Hence, in line with previous research, we find the family context to be much more important than the childhood neighbourhood context in explaining variation in adult income.

When adding time-varying control variables (Models 2b and 2d), there is no variation in adult income left to explain at the level of the childhood neighbourhood, and family level variation is substantially attenuated (to about 5% of the total variation). This change in variation confirms that the added control variables are correlated with both childhood neighbourhood context and childhood family context. Given the timing of these variables (childhood exposure/experiences must come before any adulthood characteristics), we argue that the results are likely to show a causal pattern where the childhood neighbourhood and childhood family influence later-in-life choices related to family composition and socio-economic status.

Looking at the coefficients of models 2a-d, we find that adulthood neighbourhood cumulative exposure to low-income neighbours has a negative effect on income. The models, without potential overcontrolling (models 2a and 2c), yield coefficient estimates of about -.0021. The average cumulative exposure for males is 350 (see Table 1), which corresponds to a coefficient of -.735, whereas the maximum achieved cumulative exposure is 969 (see Table 1), which corresponds to a coefficient of -2.035. When time-varying control variables are added in models 2b and 2d, the size of the coefficients for adulthood neighbourhood cumulative exposure to low-income neighbours are almost cut by half, for males and females alike.

The control variables work as expected: age is positively associated with income from work while having a father from a country outside of Sweden, especially a non-Western country, is associated with an income penalty. The effects of both age and ethnicity are stronger for females than for men. When adding the time-varying control variables in models 2b and 2d, the effect of ethnicity is substantially reduced while the coefficient for age is relatively stable. Looking at the coefficients of the time-varying control variables of models 2b and 2d we find, not surprisingly, that employment is the most important variable for explaining income from work (note that income from work also includes work-related transferences and that is estimated at a later point in time). Employment status is, of course, highly correlated between consecutive years and work experience also tends to pay off in terms of obtaining a higher income. We find that having children is positively correlated with income for males but negatively for females. A higher education level has a positive effect on income, especially for females, and people living in rented dwellings tend to have lower incomes compared with those in owner-occupied dwellings.

In the family models (models 3a-3d, with each model corresponding to 2a-2d) presented in Table 3, individual cumulative neighbourhood exposure is replaced by the two variables ‘family mean in neighbourhood exposure’ (measured on the family level) and ‘individual departure from the family mean’ (measured on the individual level). In model 3a, for same-sex male sibling pairs, we obtain estimates of family mean and individual departure of -.0024 and -.0016 respectively. The family mean coefficient is somewhat larger than the individual effect (estimated in model 2a), suggesting that the family mean variable captures more than the individual-level variable. In other words, the results suggest that the neighbourhood path *of the sibling* has an effect on an individual’s income from work. This effect is likely to be indirect, however, operating through the siblings’ joint background which includes both their shared family history and shared childhood neighbourhood.

**Table 3:** Results for real siblings, from family model, using family mean and individual departure from family mean. Dependent variable = logged income from work.

		Males								Females							
		Model 3a				Model 3b				Model 3c				Model 3d			
		Coef.	Std. Err.	Conf interval 95%		Coef.	Std. Err.	Conf interval 95%		Coef.	Std. Err.	Conf interval 95%		Coef.	Std. Err.	Conf interval 95%	
**Predictor variables**																	
Family mean sum % low-income neighbours		-0.0024	0.0002	-0.0028	-0.0020	-0.0014	0.0002	-0.0017	-0.0011	-0.0026	0.0002	-0.0031	-0.0021	-0.0016	0.0002	-0.0020	-0.0012
Individual departure from family mean		-0.0016	0.0003	-0.0021	-0.0011	-0.0007	0.0002	-0.0012	-0.0003	-0.0011	0.0003	-0.0017	-0.0005	-0.0009	0.0003	-0.0015	-0.0003
Age		0.0804	0.0091	0.0625	0.0982	0.0125	0.0074	-0.0020	0.0271	0.1285	0.0101	0.1087	0.1483	0.0112	0.0088	-0.0060	0.0285
Father's country of birth (ref = Sweden)	West	-0.3205	0.0618	-0.4417	-0.1993	-0.0701	0.0475	-0.1631	0.0229	-0.2970	0.0666	-0.4275	-0.1666	-0.0800	0.0546	-0.1870	0.0271
	East	-0.8706	0.1388	-1.1427	-0.5985	-0.3881	0.1064	-0.5967	-0.1796	-0.3601	0.1336	-0.6219	-0.0982	-0.1406	0.1095	-0.3552	0.0741
	Non-west	-1.1538	0.1132	-1.3758	-0.9319	-0.5965	0.0873	-0.7676	-0.4254	-1.5897	0.1219	-1.8288	-1.3507	-0.6689	0.1008	-0.8665	-0.4713
Live with partner (ref = single)						0.0631	0.0434	-0.0220	0.1482					0.0258	0.0393	-0.0512	0.1028
Children in household (ref = no))						0.1280	0.0434	0.0428	0.2131					-0.0799	0.0388	-0.1558	-0.0039
Education level (ref = LT12yrs)	12yrs					0.2302	0.0316	0.1683	0.2920					0.2864	0.0349	0.2180	0.3547
	13-14yrs					0.4608	0.0338	0.3945	0.5271					0.5410	0.0406	0.4614	0.6205
	15+yrs					0.6836	0.0323	0.6202	0.7470					0.8494	0.0364	0.7781	0.9208
Employed (ref = not employed)						3.8123	0.0391	3.7356	3.8889					3.1907	0.0349	3.1222	3.2592
Tenure (ref = home ownership)	cooperative					-0.0568	0.0333	-0.1220	0.0085					-0.0564	0.0398	-0.1344	0.0216
	rental					-0.1147	0.0299	-0.1732	-0.0562					-0.1309	0.0344	-0.1983	-0.0636
Constant		5.4288	0.2978	4.8452	6.0125	3.4217	0.2439	2.9436	3.8997	3.2549	0.3240	2.6199	3.8898	3.5309	0.2813	2.9795	4.0823
**Random effects parametres**																	
Childhood neighbourhood variance		0.0186	0.0194	0.0024	0.1437	0.0000	0.0000	0.0000	0.0000	0.0238	0.0220	0.0039	0.1456	0.0000	0.0000	0.0000	0.0000
Family variance		0.5575	0.0444	0.4769	0.6517	0.1156	0.0258	0.0746	0.1790	0.6733	0.0565	0.5711	0.7937	0.2113	0.0376	0.1492	0.2994
Residual		3.3805	0.0483	3.2872	3.4765	2.4226	0.0347	2.3557	2.4915	5.1098	0.0649	4.9842	5.2385	3.9404	0.0500	3.8435	4.0397
N		19706				19706				24924				24924			
Log Likelihood		-41407.799				-37129.102				-57196.046				-53089.543			

The individual departure from the family mean variable estimates the effect of the individual adult neighbourhood pathway which is unrelated to the sibship. The negative coefficient of this variable means that an individual who performs ‘better’ than their sibling (i.e. has a lower cumulative exposure to low-income neighbours) will have a higher income from work, whereas an individual who performs ‘worse’ than their sibling will earn less. To exemplify how results are affected by taking the sibship into account, we calculate the effect of the family mean and the individual departure combined and compare these to the results for individual exposure from the models in Table 2. We use male siblings and the results from the models without overcontrolling (models 2a and 3a). We have already shown that model 2a estimates the effect of individual cumulative neighbourhood exposure on income to be -0.735 for a male individual with a total mean cumulative exposure of 350. We repeat the exercise using the results from model 3a, for an individual with the same exposure (350) but who has a brother who has experienced either the minimum or maximum exposure to low-income neighbours (see Table 1 for values 50 and 969 respectively). In the brother-with-low-exposure scenario, the mean of the two brothers is 200 ((350+50)/2) and the individual departure for our individual is 150. These estimates give a total effect of about -0.7204 (very similar to the effect in model 2a). By contrast, the brother-with-high-exposure scenario has a total effect of -1.1053. Hence, our family model suggests that the poor performance of the brother, or rather the shared family and/or childhood neighbourhood characteristics that affects both the brother’s performance as well as other aspects of life, has a negative effect on income from work.

Apart from the changes in the coefficients related to neighbourhood exposure, models 3a-3d perform similarly to their individual model equivalents (models 2a-2d). The explained neighbourhood level and family level variation are almost identical, reinforcing the conclusion that only a very small proportion of the variation can be attributed to the childhood neighbourhood. By contrast, the effect of the family context is considerably more important. Adding time-varying control variables reduce family level variation substantively whereas childhood neighbourhood level variation disappears. Again, we suggest that this is due to causal effects where the variation on these levels are absorbed by the control variables. We have explored repeating the analysis for a group of siblings identified as twins. These results (not shown) generally reinforce our overall conclusions. Using twins however, who arguably are more similar than regular siblings, the family context increases in importance to explain variation in income from work. In the twin model, it explains 34% and 25% of the variation in income for males and females respectively, using the family models without over-controlling whereas any neighbourhood variation is completely lacking. Also, the size of coefficients for family mean increases, suggesting that the sibling (or family) is more important for individual outcomes for twins than for regular siblings, whereas the coefficient for individual departure decreases somewhat.

The results so far suggest that there is an independent effect of adult neighbourhood experiences on later in life income from work. The results also suggest that variation in income from work, 12–14 years after having left the parental home can, to some extent, be explained by differences in family context. The models show only a small amount of variation due to differences in childhood neighbourhood context. However, the family mean variable, which includes the joint cumulative exposure of two siblings after having left the parental home, represents everything that is shared by two siblings–including both the family context and the childhood neighbourhood context. Hence, it is still unclear to what extent family and childhood neighbourhood affect income later in life. To sort this out, we rerun all our models using the control group of the contextual siblings, the randomly paired individuals who are unrelated but originate from the same childhood neighbourhood (see Tables 4 and 5).

**Table 4:** Results for contextual siblings, from individual models, using own cumulative exposure to poverty neighbourhoods. Dependent variable = logged income from work.

		Males								Females							
		Model 4a				Model 4b				Model 4c				Model 4d			
		Coef.	Std. Err.	Conf interval 95%		Coef.	Std. Err.	Conf interval 95%		Coef.	Std. Err.	Conf interval 95%		Coef.	Std. Err.	Conf interval 95%	
**Predictor variables**																	
Total sum % low-income neighbours over 11 years		-0.0011	0.0002	-0.0015	-0.0008	-0.0011	0.0002	-0.0015	-0.0008	-0.0004	0.0002	-0.0008	0.0000	-0.0009	0.0002	-0.0013	-0.0005
Age		0.0353	0.0094	0.0168	0.0538	0.0185	0.0082	0.0025	0.0345	0.0639	0.0100	0.0443	0.0834	0.0249	0.0091	0.0072	0.0426
Father's country of birth (ref = Sweden)	West	-0.0156	0.0750	-0.1625	0.1314	0.1077	0.0638	-0.0175	0.2328	-0.0463	0.0884	-0.2196	0.1270	0.0398	0.0767	-0.1105	0.1900
	East	-0.1010	0.1741	-0.4423	0.2403	-0.0103	0.1485	-0.3014	0.2809	0.2392	0.2302	-0.2120	0.6904	0.4006	0.1990	0.0105	0.7907
	Non-west	-0.4836	0.1626	-0.8022	-0.1650	-0.4149	0.1390	-0.6873	-0.1425	-0.2739	0.1454	-0.5590	0.0112	0.1059	0.1272	-0.1433	0.3551
Live with partner (ref = single)						-0.0936	0.0505	-0.1926	0.0054					-0.0778	0.0422	-0.1605	0.0048
Children in household (ref = no))						0.0761	0.0508	-0.0235	0.1757					-0.1130	0.0421	-0.1955	-0.0305
Education level (ref = LT12yrs)	12yrs					0.0906	0.0363	0.0196	0.1617					0.0930	0.0384	0.0177	0.1683
	13-14yrs					0.1680	0.0385	0.0926	0.2435					0.2123	0.0437	0.1267	0.2979
	15+yrs					0.4030	0.0370	0.3304	0.4756					0.4037	0.0384	0.3284	0.4791
Employed (ref = not employed)						1.1847	0.0537	1.0796	1.2899					0.9228	0.0430	0.8384	1.0071
Tenure (ref = home ownership)	cooperative					0.0293	0.0389	-0.0470	0.1055					0.0184	0.0430	-0.0660	0.1027
	rental					-0.1132	0.0347	-0.1813	-0.0452					-0.0291	0.0367	-0.1011	0.0428
Constant		6.8512	0.3095	6.2446	7.4578	6.1764	0.2741	5.6391	6.7138	5.2992	0.3213	4.6695	5.9289	5.8110	0.2906	5.2414	6.3806
**Random effects parametres**																	
Childhood neighbourhood variance		0.0000	0.0000	0.0000	0.0000	0.0000	0.0000	0.0000	0.0000	0.0093	0.0141	0.0005	0.1819	0.0000	0.0000	0.0000	0.0000
Family variance		0.0000	0.0000	0.0000	0.0000	0.0000	0.0000	0.0000	0.0000	0.0000	0.0000	0.0000	0.0000	0.0000	0.0000	0.0000	0.0000
Residual		0.4181	0.0141	0.3913	0.4468	0.3007	0.0102	0.2814	0.3213	0.5086	0.0209	0.4693	0.5513	0.3928	0.0122	0.3696	0.4174
N		1750				1750				2082				2082			
Log Likelihood		-1720.1443				-1431.7204				-2269.1244				-1981.435			

**Table 5:** Results for contextual siblings, from family model, using family mean and individual departure from family mean. Dependent variable = logged income from work.

		Males								Females							
		Model 5a				Model 5b				Model 5c				Model 5d			
		Coef.	Std. Err.	Conf interval 95%		Coef.	Std. Err.	Conf interval 95%		Coef.	Std. Err.	Conf interval 95%		Coef.	Std. Err.	Conf interval 95%	
**Predictor variables**																	
Family mean sum % low-income neighbours		-0.0012	0.0002	-0.0017	-0.0008	-0.0011	0.0002	-0.0016	-0.0007	-0.0003	0.0003	-0.0009	0.0002	-0.0009	0.0002	-0.0013	-0.0004
Individual departure from family mean		-0.0010	0.0003	-0.0015	-0.0005	-0.0011	0.0002	-0.0016	-0.0007	-0.0004	0.0003	-0.0010	0.0002	-0.0009	0.0003	-0.0015	-0.0004
Age		0.0353	0.0094	0.0168	0.0538	0.0185	0.0082	0.0025	0.0345	0.0639	0.0100	0.0443	0.0834	0.0249	0.0091	0.0072	0.0426
Father's country of birth (ref = Sweden)	West	-0.0159	0.0750	-0.1628	0.1310	0.1076	0.0638	-0.0175	0.2328	-0.0464	0.0884	-0.2197	0.1269	0.0397	0.0767	-0.1105	0.1900
	East	-0.0930	0.1745	-0.4351	0.2491	-0.0094	0.1489	-0.3013	0.2824	0.2393	0.2302	-0.2119	0.6906	0.4009	0.1990	0.0109	0.7910
	Non-west	-0.4801	0.1626	-0.7988	-0.1613	-0.4145	0.1391	-0.6871	-0.1419	-0.2755	0.1469	-0.5634	0.0124	0.1020	0.1284	-0.1497	0.3537
Live with partner (ref = single)						-0.0938	0.0505	-0.1928	0.0053					-0.0777	0.0422	-0.1603	0.0050
Children in household (ref = no))						0.0762	0.0508	-0.0234	0.1758					-0.1132	0.0421	-0.1957	-0.0307
Education level (ref = LT12yrs)	12yrs					0.0907	0.0363	0.0196	0.1617					0.0928	0.0384	0.0175	0.1681
	13-14yrs					0.1681	0.0385	0.0926	0.2436					0.2123	0.0437	0.1267	0.2979
	15+yrs					0.4030	0.0370	0.3304	0.4756					0.4037	0.0384	0.3284	0.4791
Employed (ref = not employed)						1.1846	0.0537	1.0794	1.2898					0.9228	0.0430	0.8384	1.0071
Tenure (ref = home ownership)	cooperative					0.0292	0.0389	-0.0470	0.1055					0.0184	0.0430	-0.0659	0.1028
	rental					-0.1133	0.0347	-0.1813	-0.0452					-0.0288	0.0367	-0.1008	0.0432
Constant		6.8867	0.3142	6.2709	7.5025	6.1803	0.2783	5.6348	6.7258	5.2948	0.3264	4.6551	5.9344	5.8005	0.2945	5.2232	6.3778
**Random effects parametres**																	
Childhood neighbourhood variance		0.0000	0.0000	0.0000	0.0000	0.0000	0.0000	0.0000	0.0000	0.0093	0.0141	0.0005	0.1817	0.0000	0.0000	0.0000	0.0000
Family variance		0.0000	0.0000	0.0000	0.0000	0.0000	0.0000	0.0000	0.0000	0.0000	0.0000	0.0000	0.0000	0.0000	0.0000	0.0000	0.0000
Residual		0.4180	0.0141	0.3912	0.4467	0.3007	0.0102	0.2814	0.3213	0.5086	0.0209	0.4693	0.5513	0.3928	0.0132	0.3678	0.4195
N		1750								2082				2082			
Log Likelihood		-1719.9309				-1431.7172				-2269.1213				-1981.4109			

Two features of the contextual sibling models (4a-d for individual models, and 5a-d for family models) are specifically noteworthy. First, the results show that the contextual sibling design works as expected. The family variance in the models is zero, as it should be for unrelated individuals who do not share the same family context. Second, the family models with the contextual siblings (models 5a-5d) yield lower coefficient values for the family mean variable compared to the models with the real siblings (models 3a-3d). This probably means that the part of the coefficient that captures the shared family context is absent, which also makes sense as these are not real but contextual siblings. So the models for contextual siblings show that the models for the real siblings are able to capture family context effects. For the contextual siblings we repeat the exercise of calculating the joint effect of family mean and individual departure from the mean for a hypothetical individual with a mean exposure (of 350) and a ‘sibling’ with low (50) or high (969) exposure. We find that for the real siblings the overall neighbourhood effects are much stronger than for the contextual siblings. We suggest this can be explained by family context effects. For the real siblings we also capture a family effect. Also, we find that for the contextual siblings the effect of having a high poverty exposure sibling is 26% higher than the effect of having a low exposure sibling, while in the model for real siblings this is 53%. We also interpret this difference as a family context effect which is present for the real siblings, but not for the contextual siblings.

Interestingly, in the contextual siblings models the childhood neighbourhood variance is zero. By design, the contextual siblings do share their childhood neighbourhood so any (causal) effects from the childhood neighbourhood on later-in-life income from work should be captured on the neighbourhood level. That we do not find this suggests, contrary to several previous studies, that for this population the childhood neighbourhood has no long-lasting significant effect on income from work. Using real siblings (models 2a and c, and models 3a and c), we did find (a very small) variance to be explained on the neighbourhood level. We suggest that the childhood neighbourhood variance found in these models were actually related to the family context, and the fact that families sort into specific neighbourhoods. Had it really been a neighbourhood effect, we would also find it in the contextual sibling models.

## Summary

This paper set out to better understand the effects of childhood neighbourhood context, and adulthood neighbourhood experiences on individual income from work later in life. The paper started with the idea that estimation of these neighbourhood effects is likely to be affected by the influence of the childhood family context. The childhood family sorts children into certain childhood neighbourhoods, affects adult neighbourhood careers, but also affects later in life income from work. Separating these different effects is a major challenge in neighbourhood effects research, because any childhood family effect might bias estimates of independent causal effects on income of childhood and adult neighbourhood experiences.

In this study we sought to overcome the family contextual bias by using a sibling design, supplemented with analyses for contextual sibling pairs as controls. These contextual siblings do not share the childhood family, but do share childhood neighbourhood experiences. Comparing analyses for real siblings and contextual siblings can give greater insight into the different mechanisms at play. The overall results suggest that adult neighbourhood experiences do affect later in life income from work, but that there is no meaningful effect of the childhood neighbourhood context. However, the childhood family context is important in explaining later in life outcomes.

These conclusions were derived from four sets of analyses (two for real siblings and two for contextual siblings). We first modelled the effect of individual level neighbourhood experiences on income from work for real siblings (Table 2). The results suggest that longer term exposure to high poverty neighbours has a negative effect on income from work. However, this model cannot separate this effect from the effect of the childhood neighbourhood and the childhood family context and they may therefore be confounded. Our individual level model shows that there is little variance to explain at the level of the childhood neighbourhood, and that the childhood family context is much more important in understanding income. The family model for the real siblings shows a family mean effect on income later in life, which is likely to be a combination of the childhood family and childhood neighbourhood context effects. The family model for the real siblings also shows an individual departure from the family mean effect; this can be interpreted as an effect of adult neighbourhood experiences on income from work. The model leaves no childhood neighbourhood variance to explain, but explains the family level variance reasonably well.

Next we ran models for the contextual siblings (See Tables 4 and 5). These models were designed to test if the sibling design works, and to assess whether there is an effect of the childhood neighbourhood context on income from work. The models for the contextual siblings show that indeed the sibling setup works well as the sibling models explain nothing at the level of the family, which makes sense as contextual siblings are not real siblings by design. Interestingly, the contextual sibling models also have no variance to explain at the level of the childhood neighbourhood context. This suggests that there is no childhood neighbourhood effect on later in life income from work. The contextual sibling model does show an effect of adulthood neighbourhood experiences on income.

## Conclusion

The results suggest that there is an adulthood neighbourhood effect on income from work, net of the effect of the childhood neighbourhood and childhood family context effects. The results also suggest that any effects on later in life income from the childhood neighbourhood context are in fact childhood family context effects. That is not to say that the childhood neighbourhood is not important at all, but likely that the childhood neighbourhood effect is the result of non-random selection of families into neighbourhoods based on family characteristics. Our analyses show that individuals with a sibling who does well in terms of their (adult) neighbourhood pathway (in other words has a low cumulative exposure to low-income neighbourhoods), have a higher predicted income from work compared to individuals with a sibling with a high exposure to low-income neighbourhoods. We interpret this as a family context effect. Those with siblings in low income neighbourhoods are assumed to come from a less resourceful or advantageous family (either in terms of finances, time investments or other unobservable but important traits such as genetics), whereas individuals whose siblings live in better neighbourhoods are assumed to benefit from a more positive family background. Our overall conclusion, therefore, is that the childhood family context has a lasting effect on adult income, even when taking both childhood and adult neighbourhood path into account. Part of what appeared to be a neighbourhood effect was in fact a lasting ‘family effect’. For the wider research literature, it is clear that, when possible, models of neighbourhood effects should control for the childhood family context to avoid bias in estimates.

## Discussion

A possible limitation of our study is the construction of the contextual sibling pairs. Because of pragmatic and conceptual restrictions we have used a relatively simple way to construct a control group of contextual siblings. Although we had access to full population data, imposing more restrictions on the contextual siblings would reduce the size of the control group further. A larger control group could be constructed in countries with larger populations, or by using multiple cohorts within the data. A further limitation is that the real sibling pairs differ in ways we cannot observe in the data. To reduce these possible differences, a dataset of real (preferably identical) twins could be used, but that requires a dataset with a large number of twins, requiring at least a birth cohort study or preferably a twin study. In these cases we would likely be able to acquire genetic information as well allowing further control of currently unobservable factors. However, using our design, we got the most out of the register data at our disposal.

This study contributes to current debates in the neighbourhood effects literature on differential impacts of similar neighbourhood environments on different people (see [23] and [52]). We add to the discussion of individual heterogeneity by arguing that the overall effect may differ among individuals depending on the characteristics of their parental family background and former neighbourhood experiences. Although the family background is not deterministic in any sense–for instance, individuals may perform well despite coming from a less advantageous family background, or do relatively badly in terms of neighbourhood path despite having a resourceful family–the childhood family context generally has a lasting effect on individual income later in life. These results were acquired using data from Sweden, a country that provides relatively good opportunities for individuals to ‘move up’ on the social ladder in terms of both income and neighbourhood path. Although there is indeed a link between family background and individual performance (see [44] on socio-economic status; [4] on neighbourhood status), it should be easier to undertake upward social mobility in terms of neighbourhood status in counties characterized by relatively high levels of income equality, such as Sweden, than in more liberal welfare regimes. Hence, it is likely that the ‘family effects’ found in this paper are stronger in other types of societies.
